# Glucagon-like peptide-1 and glucagon-like peptide-2 regulation during human liver regeneration

**DOI:** 10.1038/s41598-023-43283-8

**Published:** 2023-09-25

**Authors:** Markus Ammann, Jonas Santol, David Pereyra, Tamara Kalchbrenner, Tanja Wuerger, Johannes Laengle, Rory L. Smoot, Wolfgang Hulla, Friedrich Laengle, Patrick Starlinger

**Affiliations:** 1 Department of Surgery, State Hospital Wiener Neustadt, Wiener Neustadt, Austria; 2 Division of Visceral Surgery, Department of General Surgery, Medical University of Vienna, Vienna, Austria; 3 Department of Surgery, HPB Centre, Viennese Health Network, Clinic Favoriten and Sigmund Freud Private University, Vienna, Austria; 4 Department of Pathology, State Hospital Wiener Neustadt, Wiener Neustadt, Austria; 5 Department of Surgery, Division of Hepatobiliary and Pancreas Surgery, Mayo Clinic, 200 First Street SW, Rochester, MN USA

**Keywords:** Medical research, Translational research, Hepatology

## Abstract

Accumulating evidence suggests that metabolic demands of the regenerating liver are met via lipid metabolism and critical regulators of this process. As such, glucagon-like peptide-1 (GLP-1) and glucagon-like peptide-2 (GLP-2) critically affect hepatic regeneration in rodent models. The present study aimed to evaluate potential alterations and dynamics of circulating GLP-1 and GLP-2 in patients undergoing liver resections, focusing on post-hepatectomy liver failure (PHLF). GLP-1, GLP-2, Interleukin-6 (IL-6) and parameters of lipid metabolism were determined perioperatively in fasting plasma of 46 patients, who underwent liver resection. GLP-1 and GLP-2 demonstrated a rapid and consistently inverse time course during hepatic regeneration with a significant decrease of GLP-1 and increase of GLP-2 on POD1. Importantly, these postoperative dynamics were significantly more pronounced when PHLF occurred. Of note, the extent of resection or development of complications were not associated with these alterations. IL-6 mirrored the time course of GLP-2. Assessing the main degradation protein dipeptidyl peptidase 4 (DPP4) no significant association with either GLP-1 or -2 could be found. Additionally, in PHLF distinct postoperative declines in plasma lipid parameters were present and correlated with GLP-2 dynamics. Our data suggest dynamic inverse regulation of GLP-1 and GLP-2 during liver regeneration, rather caused by an increase in expression/release than by changes in degradation capacity and might be associated with inflammatory responses. Their close association with circulating markers of lipid metabolism and insufficient hepatic regeneration after liver surgery suggest a critical involvement during these processes in humans.

## Introduction

The regenerative capacity of the liver remains the single most relevant factor determining postoperative outcome after hepatic resection, as post-hepatectomy liver failure (PHLF) remains the most frequent cause of mortality after partial hepatectomy^1^.

The energetically highly demanding process of hepatic regeneration requires vast changes in systemic energy distribution^2^. Accumulating evidence suggests that metabolic demands of the regenerating liver are primarily met via lipid metabolism. In fact, partial hepatectomy induces a rapid postoperative decline of lean- and adipose tissue mass paralleled by a concomitant increase in accumulation of lipids in the regenerating liver in rodents, leading to transient steatosis^2,3^. Intriguingly, the predominant proportion of newly synthesized adenosine triphosphate (ATP) during liver regeneration is derived from β-oxidation^4^ and regeneration is impaired when intrahepatic lipid accumulation is disrupted^3^. However, the signals responsible for intrahepatic lipid accumulation are still poorly understood^2^.

In this context, glucagon-like peptide-2 (GLP-2) and its sister molecule glucagon-like peptide-1 (GLP-1) are of particular interest, as being critically involved in hepatic lipid homeostasis^5,6^. In particular, GLP-2 increases fasting plasma triglyceride level, very low-density lipoprotein (VLDL) production and hepatic lipogenesis, thereby leading to hepatic steatosis^7^. GLP-1 has opposing effects on hepatic lipid accumulation and plasma lipid levels, which has recently broadened its clinical use in pharmacological therapy of metabolic syndrome and steatohepatitis^8,9^. Of note, significant effects of GLP-2 and GLP-1 on hepatic regeneration have been described in rodent models of partial hepatectomy, suggesting a pro-regenerative role of GLP-2^10^, whereas GLP-1 seems to deteriorate post-hepatectomy liver regeneration^11^.

The short half-life of active GLP-1 and GLP-2 of only a few minutes due to rapid degradation by dipeptidyl peptidase-4 (DPP4)^12^ suggests a rather dynamic regulation of these proteins. While human data on pathophysiological regulation of these proteins is largely missing, Interleukin-6^13^ and bile acids^14^ were identified as stimulators besides the “classic” L-cell activators. Their time course and regulation during human liver regeneration remains unknown.

Given the documented effects of GLP-1 and GLP-2 on rodent liver regeneration and their routine therapeutic targeting for other indications, we sought to explore perioperative dynamics of GLP-1 and GLP-2 in patients undergoing hepatic resections. We further aimed for comparison of perioperative dynamics of GLP-1 and GLP-2 in the context of impaired liver regeneration of patients developing PHLF. Ultimately, we aimed to generate explorative evidence on how their levels might be regulated and their associations with circulating lipids in the setting of human liver regeneration.

## Results

### Patient demographics

To study perioperative GLP-1 and GLP-2 plasma concentration dynamics, we included 46 patients, undergoing minor (n = 26) or major (n = 20) hepatic resections. Twenty patients had hepatic resection for metastasized colorectal carcinoma (mCRC), 10 for hepatocellular carcinoma (HCC), 12 for intrahepatic or perihilar cholangiocarcinoma carcinoma (iCCA/pCCA), 1 for neuroendocrine liver metastasis (NELM), 1 for non-neuroendocrine non-colorectal (NNECR) liver metastasis and 2 patients had benign liver lesions. Seven patients developed PHLF, of whom 3 died from liver failure. Patient demographics are shown in Table 1.

**Table 1:** Patient demographics and clinical data.

	NoPHLF		PHLF		p-value
	N (%)/median (range)		N (%)/median (range)		
Age (years)		65 (33–84)		67 (61–79)	
Sex					
Male		26 (67)		5 (71)	
Female		13 (33)		2 (29)	
Diabetes mellitus type II		8 (5)		4 (57)	
DPP4 inhibitor treatment		3 (37)		3 (75)	
BMI (kg/m^2^)		27.3 (18.6–41.0)		27.5 (24.0–37.9)	
Tumour type					
mCRC		20 (51)		0 (0)	
HCC		8 (21)		2 (29)	
iCCC/pCCA		7 (18)		5 (71)	
Other		2 (5)		0 (0)	
Benign		2 (5)		0 (0)	
Preoperative chemotherapy		20 (51)		0 (0)	
Resection extend					
Minor		26 (67)		0 (0)	
Major		13 (33)		7 (100)	
Preoperative liver augmentation		3 (8)		3 (75)	
Morbidity ≥ 3		5 (13)		7	
Perioperative mortality		0 (0)		3 (75)	
Blood parameters^†^					
CRP (mg/dl)	39	0.4 (0.0–17.2)	7	0.9 (0.1–14.4)	0.049
IL-6 (pg/ml)	37	5.0 (0.00–52.60)	6	10.8 (0.0–72.3)	0.344
Bile acids (µmol/l)	35	3.6 (0.10–53.60)	6	5.9 (2.2–20.1)	0.196
Bilirubin (mg/dl)	39	0.5 (0.1–8.7)	7	0.6 (0.2–5.1)	0.697
Platelets (G/L)	39	231.0 (99–403)	7	191,0 (127–438)	0.905
Prothrombin time (%)	39	99.0 (71–114)	7	92,0 (75–108)	0.183
Antithrombin III (%)	37	85.0 (34–120)	7	81.0 (41–99)	0.469
Triglycerides (mg/dl)	39	114.0 (49–534)	7	164.0 (89–343)	0.145
Cholesterol (mg/dl)	39	190.0 (97–340)	7	159.0 (86–209)	0.066
LDLc (mg/dl)	38	106.5 (32–186)	6	71.0 (28–136)	0.098
HDLc (mg/dl)	38	50.5 (15–103)	6	31.5 (24–65)	0.030
ApoB (g/l)	39	1.0 (0.43–1.89)	6	0.78 (0.56–1.20)	0.591
ApoA1 (g/l)	39	1.37 (0.15–2.06)	6	1.11 (0.52–1.57)	0.030
γ-Glutamyltransferase (U/l)	39	54 (9–712)	7	181 (64–499)	0.007
Aspartate aminotransferase (U/l)	39	30 (13–73)	7	45 (9–94)	0.529
Alanine aminotransferase (U/l)	39	24 (8–425)	7	69 (7–214)	0.128
Alkaline phosphatase (U/l)	39	83 (40–594)	7	154 (63–458)	0.023

Values in parenthesis represent percentages unless indicated otherwise; ^†^values represent medians(range). *DPP4* dipeptidyl peptidase-4, *BMI* body mass index, *mCRC* metastasized colorectal carcinoma, *HCC* hepatocellular carcinoma, *iCCA/pCCA* intrahepatic/perihilar cholangio carcinoma, *IL-6* Interleukin-6, *LDLc* low-density lipoprotein cholesterol, *HDLc* high-density lipoprotein cholesterol, *ApoB* apolipoprotein B, *ApoA1* apolipoprotein A1, Mann–Whitney-*U* test. Significance level p < 0.005.

### GLP-2 plasma levels increase postoperatively, but not conditionally upon resection extent

First, we wanted to evaluate if GLP-1 and GLP-2 plasma concentrations in patients are altered upon liver resection (LR). Additionally, we aimed to explore possible perioperative dynamics and if differences are related to the extent of lost liver tissue. Therefore, we excluded patients who developed PHLF and/or postoperative morbidity (Dindo ≥ 3) and explored the influence of the resection extent (major LR vs. minor LR) on GLP-1 and GLP-2 plasma concentration dynamics and compared plasma levels at perioperative timepoints.

**Figure 1:**
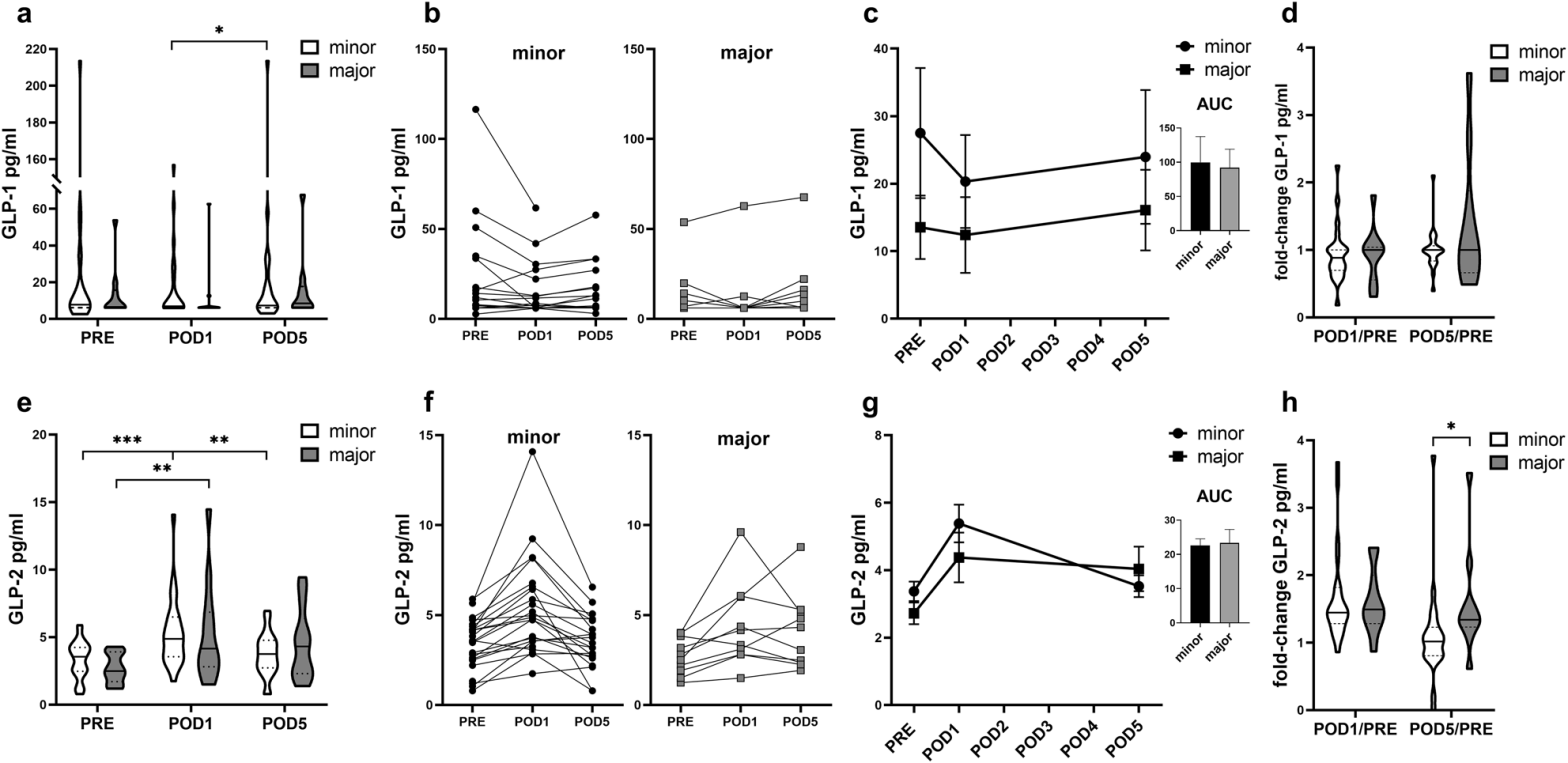
Perioperative GLP-1 and GLP-2 plasma concentrations in liver resections respective to resection extend. PHLF and morbidity ≥ 3 excluded; Violine blots with horizontal and dotted lines for means and quartiles. Concentrations at timepoints are displayed and changes between timepoints indicated (**a**, **e**). Individual perioperative dynamics regarding resection extent are shown (**b**, **f**). Graphs with error-bars illustrate perioperative GLP dynamics and columns depict AUC comparisons (**c**, **g**). Changes in GLP-1 and GLP-2 plasma level until POD5 are expressed as fold-change compared to baseline level preoperatively (**d**, **h**); Mann–Whitney-*U* test; Wilcoxon signed-rank test; *p < 0.05; **p < 0.005; ***p < 0.0005.

GLP-1 plasma levels did not change according to baseline concentrations following liver surgery, regardless of the resection extent. Although in minor LR GLP-1 level decreased from POD1 to POD5, but between minor and major LR no differences could be observed on any perioperative timepoints (Fig. 1a–d, Supplementary Materials & Methods Table S1). Remarkably, GLP-2 levels increased 1.9-fold in both, minor and major LR, within the first postoperative day. In minor LR GLP-2 reached baseline level until POD5, whereas in major LR GLP-2 level elevation remained (Fig. 1e–h). Taken these findings together, an early postoperative, physiological increase of GLP-2 plasma concentrations in all patients can be observed, regardless of the resected liver volume, indicating an upregulation during liver regeneration, timely persisting in resections of greater extent.

### PHLF is associated with decreased GLP-1 and increased GLP-2 plasma concentrations

**Figure 2:**
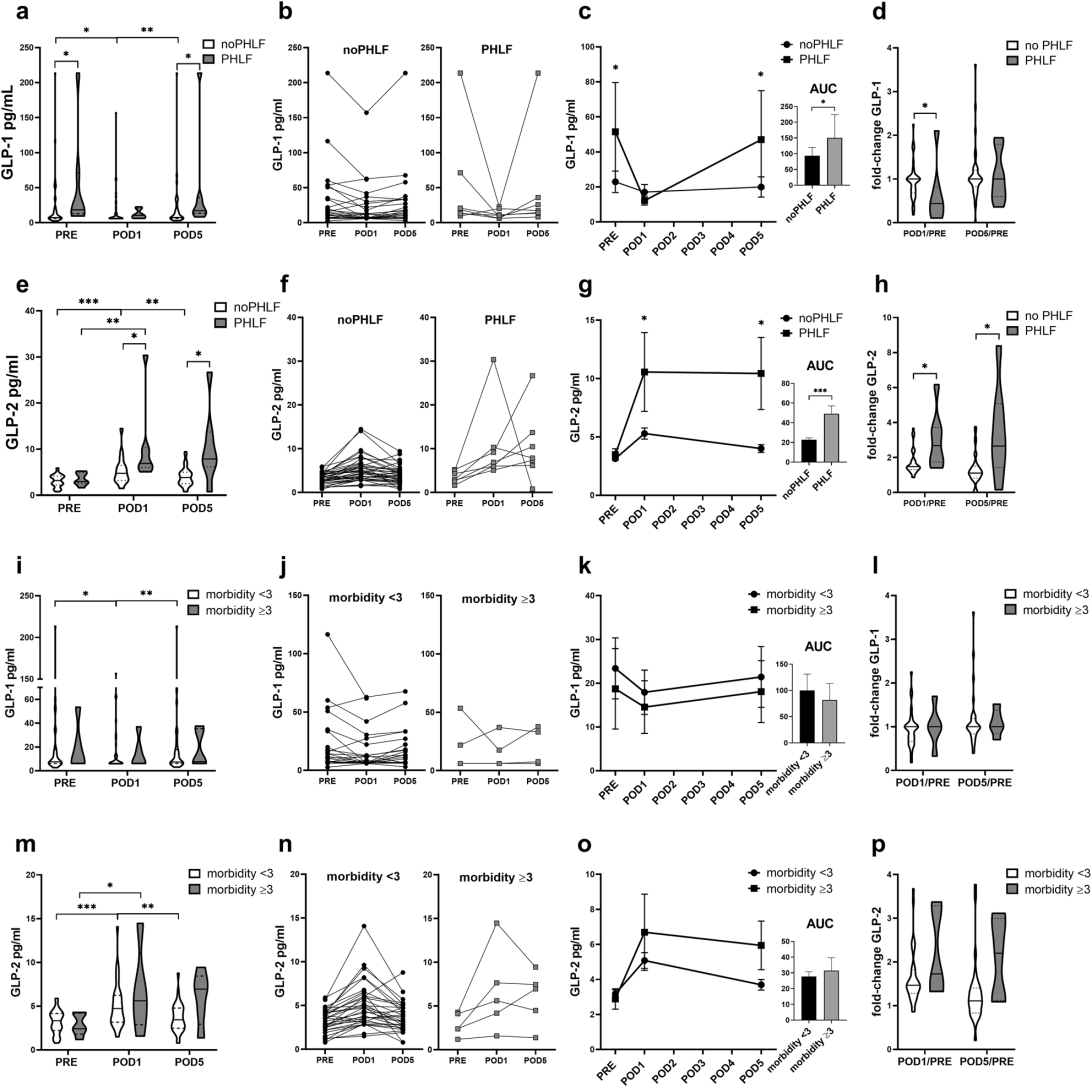
Perioperative GLP-1 and GLP-2 concentrations in the total cohort regarding PHLF and in postoperative morbidity when PHLF was excluded; Differences in GLP-1 and GLP-2 plasma levels between groups and perioperative timepoints are indicated (**a**, **e**, **i**, **m**). Individual perioperative dynamics are shown (**b**, **f**, **j**, **n**). Graphs with error-bars illustrate perioperative GLP level dynamics, columns with error- bars exemplify differences in the AUCs (**c**, **g**, **k**, **o**). Postoperative changes of GLP-1 and GLP-2 plasma level until POD5 are depicted as fold-change compared preoperative values (**d**, **h**, **l**, **p**); Man-Whitney-*U* test; Wilcoxon signed-rank test; *p < 0.05; **p < 0.005; ***p < 0.0005.

Next, we focused on perioperative GLP-1 and GLP-2 plasma concentration dynamics in patients who developed PHLF. We observed, that patients who developed PHLF displayed 2.3-fold higher GLP-1 plasma concentrations preoperatively, compared to patients without PHLF (*p* = 0.030). Further a trend towards a postoperative decline until POD1 was present in both, but 3.2-times more pronounced in patients who developed PHLF (p = 0.027) (Fig. 2a–c, Supplementary Materials & Methods Table S2). Until POD5, GLP-1 level of patients with PHLF seem to recover to baseline levels (Fig. 2c,d). Although, given the pronounced early postoperative GLP-1 level alterations in PHLF, the AUC of GLP-1 plasma level trajectories, were similar (*p* = 0.114) (Fig. 2c, Supplementary Materials & Methods Table S3).

In contrast, preoperative GLP-2 plasma concentrations did not differ, but increased 3.4-fold within the first postoperative day in patients with PHLF, compared to an 1.5-times increase in patients who did not develop PHLF, resulting in 2.0-times higher GLP-2 level in PHLF on POD1 (*p* = 0.008). This difference became even more apparent, as in patients without PHLF, GLP-2 levels decreased until POD5 but remained 2.6-fold higher in patients who developed PHLF (*p* = 0.006) (Fig. 2e–h, Supplementary Materials & Methods Table S2). This dynamic could be further illustrated in a significantly greater AUC (*p* < 0.001) and GLP-2 level ratios at specific timepoints compared to baseline in patients who developed PHLF (Fig. 2g + h, Supplementary Materials & Methods Table S3).

These results not only show a disconnect between GLP-1 and GLP-2 dynamics in the early postoperative period, as further illustrated by the absence of correlations at perioperative time points (Supplementary Materials & Methods Fig. S1), but also indicate a persistent up-regulation of GLP-2 in the context of PHLF.

### Perioperative GLP-1 and GLP-2 dynamics are not associated with postoperative morbidity

Assuming PHLF to be a discrete entity, which secondary leads to morbidity and mortality, we aimed to evaluate, if differences in perioperative GLP concentration dynamics are caused by postoperative complications independently of PHLF. Therefore, we excluded all PHLF patients and compared GLP-1 and GLP-2 plasma levels between patients with morbidity ≥ 3 and patients with an uneventful postoperative course.

In the postoperative course, we did not observe significant dynamics of GLP-1 concentrations in patients with severe morbidity, neither were GLP-1 plasma concentration differences present on respective timepoints (Fig. 2i–l). On the other hand, postoperative GLP-2 concentrations increased in both groups, remaining elevated in patients with severe postoperative morbidity, while in patients with an uneventful postoperative course, the levels further decreased to baseline. No significant differences were observed in the trajectories of GLP-2 plasma concentrations or at respective time points (Fig. 2m–p, Supplementary Materials & Methods Table S4). Summarizing these results, it is conceivable, that postoperative GLP-1 and GLP-2 dynamics are not solely a mechanism in the context of severe postoperative complications but a feature of a dysfunctional regeneration of the liver itself.

### GLP-1 but not GLP-2 baseline plasma level are associated with obesity, and histological features of MASLD

Given to previous reports that GLP plasma concentrations are altered in obesity and MASLD, which are frequently associated with diabetes, we therefore thought to investigate whether baseline GLP-1 or GLP-2 plasma level are associated with these conditions. Patients with BMC ≥ 25 kg/m^2^ presented higher GLP-1 (p = 0.041), but not GLP-2 levels (Supplementary Materials & Methods Fig. S2b). Similar alterations were observed in histopathological findings of steatosis (p = 0.004) and inflammatory activity (p = 0.040) (Supplementary Materials & Methods Fig. S2c + d). The presence of fibrosis of any stage was not associated with differences in GLP-1 and GLP-2 levels (Supplementary Materials & Methods Fig. S2e). Patients with MASLD on the other hand, rendered a non-significant trend towards elevated preoperative GLP-1 and GLP-2 levels. Importantly, no associations of GLP-1 and GLP-2 concentrations at baseline were seen regarding underlying diabetes mellitus (Supplementary Materials & Methods Fig. S2a). These findings indicate, that patients who suffer from obesity or an underlying liver disease, displayed higher preoperative GLP-1 plasma levels, whereas GLP-2 levels do not appear to be affected.

### Postoperative GLP-1/GLP-2 concentrations appear production controlled and not degradation associated

Given previous reports of bile acids and IL-6 having profound effects on GLP secretion of enteroendocrine L-cells, our next focus was on dynamics of plasma bile acids and IL-6 concentrations, particularly their perioperative dynamics.

**Figure 3:**
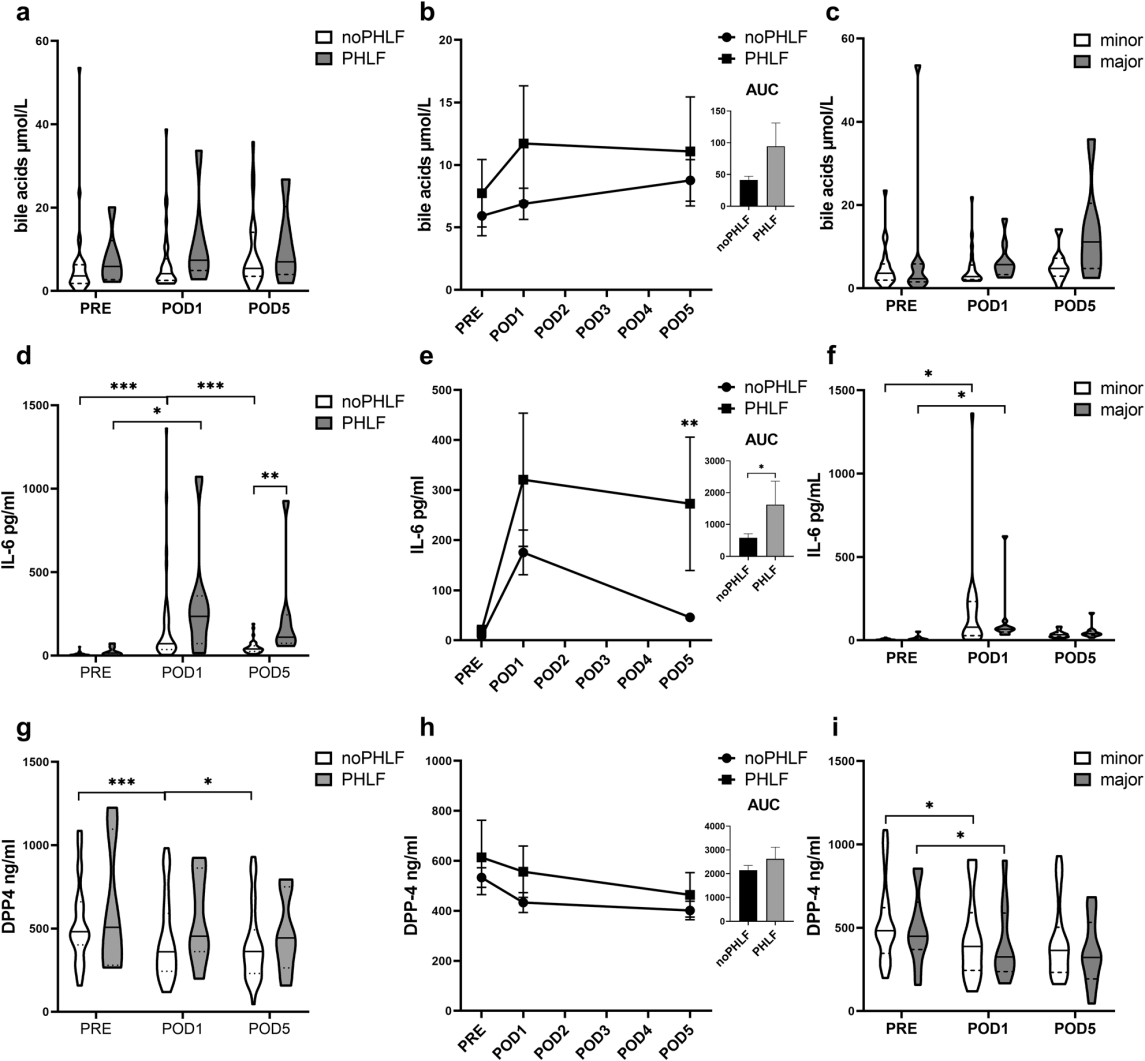
Perioperative bile acids, IL-6 and DPP4 plasma levels. Comparison of bile acids (**a**), IL-6 (**d**) and DPP4 (**g**) plasma concentrations regarding PHLF on and between perioperative timepoints. Violine blots with horizontal and dotted lines for means and quartiles. Dynamics illustrated in graphs with error- bars and comparison of AUCs displayed by columns with error bars (**b**, **e**, **h**). Postoperative differences of bile acid (**c**), IL-6 (**f**) and DPP4 (**i**) concentrations associated with the resection extent solely (major vs. minor resections, PHLF excluded), are displayed between and on specific timepoints. Mann–Whitney-*U* test; Wilcoxon signed-rank test; *IL-6* Interleukin-6, *DPP4* dipeptidyl peptidase 4, *p < 0.05; **p < 0.005; ***p < 0.0005.

We observed no difference in plasma bile acid concentrations between patients with and without PHLF. Neither a difference at indicated timepoints, nor dynamics between timepoints or patients were observed (Fig. 3a–c). Furthermore, when examining the influence of the extent of resection on changes in bile acid concentrations, after excluding patients who developed PHLF or experienced morbidity of ≥ 3, we did not observe any differences in bile acid concentrations or their postoperative dynamics (Fig. 3c).

IL-6 levels exhibited an early postoperative increase in both groups, with no differences regarding PHLF development (Fig. 3d). In the further postoperative time course, IL-6 level remained elevated in PHLF whereas in patients without PHLF, IL-6 decreased, resulting in a pronounced elevation of IL-6 in PHLF on POD5 (*p* < 0.001). These dynamics were also mirrored by a significant difference in the AUC of perioperative IL-6 trajectories (*p* = 0.005) (Fig. 3d + e). Once again, after excluding patients with PHLF or morbidity ≥ 3, no disparities in postoperative IL-6 levels associated with the extent of resected liver tissue were apparent (Fig. 3f). These results indicated, that impaired liver regeneration rather than the resection extent is associated with elevated postoperative IL-6 levels.

DPP4 is highly expressed in the liver, but, as our observation of immunohistochemical DPP4 staining revealed, mainly located on the biliary compartment of hepatocytes and not in the sinusoids (Supplementary Materials & Methods and Fig. S3). Given the fact that the soluble enzymatically active form of DPP4 might be responsible for the extensive degradation of GLP-1 and GLP-2 in the portal circulation, we sought to evaluate if changes in circulating DPP4 levels are associated with distinct GLP-1 or GLP-2 dynamics in PHLF. We did not observe any differences in DPP4 levels at any timepoints, nor did we notice any remarkable dynamics within the first 5 postoperative days in patients with PHLF (Fig. 3g,h). After excluding patients with PHLF and morbidity ≥ 3, we compared DPP4 dynamics according to extent of resected liver volume. We could observe an initial postoperative decrease of circulating DPP4 level in both, minor and major resections, but no differences at certain timepoints (Fig. 3i). Summarized, these findings point out, that DPP4 levels are somehow affected during the postoperative course, but hepatic prone soluble DPP4 might not be primarily responsible for decreased plasma concentrations, due to the circumstance that declines are not associated with the extent of resected liver volume.

We next evaluated for a possible association between bile acid, IL-6 or circulating DPP4 levels and the plasma GLP-1 and GLP-2 concentrations in the perioperative course. Preoperatively, a weak correlation of bile acids could be observed for both, GLP-1 and GLP-2. Postoperatively IL-6 levels showed a more pronounced association with GLP-1 and GLP-2 concentrations. GLP-2 levels on the other hand seemed to correlate at any timepoint with IL-6, even more distinct postoperatively. DPP4 showed no association with either GLP-1 or GLP-2 at any timepoint. (Table 2, Supplementary Materials & Methods Fig. S4).

**Table 2:** Spearman corelations of DPP4, IL-6 and bile acids levels with GLP-1 and GLP-2 level at perioperative timepoints.

	GLP-1			GLP-2		
	n	p-value	R	n	p-value	R
Bile acids						
PRE	41	0.017	0.370	41	0.048	0.311
POD1	41	0.105	0.257	41	0.087	0.270
POD5	31	0.241	0.217	31	0.426	0.148
IL-6						
PRE	43	0.073	0.276	43	0.031	0.330
POD1	44	0.031	0.327	44	< 0.001	0.517
POD5	39	0.242	0.192	39	< 0.001	0.582
DPP4						
PRE	46	0.078	− 0.263	46	0.547	− 0.091
POD1	45	0.957	0.008	45	0.202	0.194
POD5	43	0.562	0.091	43	0.198	0.200

*DPP4* dipeptidyl peptidase-4, *IL-6* Interleukin-6, *GLP-1* glucagon like peptide-1, *GLP-2* glucagon-like peptide-2.

### PHLF and the GLP-1/GLP-2 ratio are associated with distinct postoperative plasma lipid dynamics

Given previous evidence, that lipid metabolism is altered during liver regeneration we aimed to study perioperative changes of clinical routine lipid parameters in liver regeneration.

**Figure 4:**
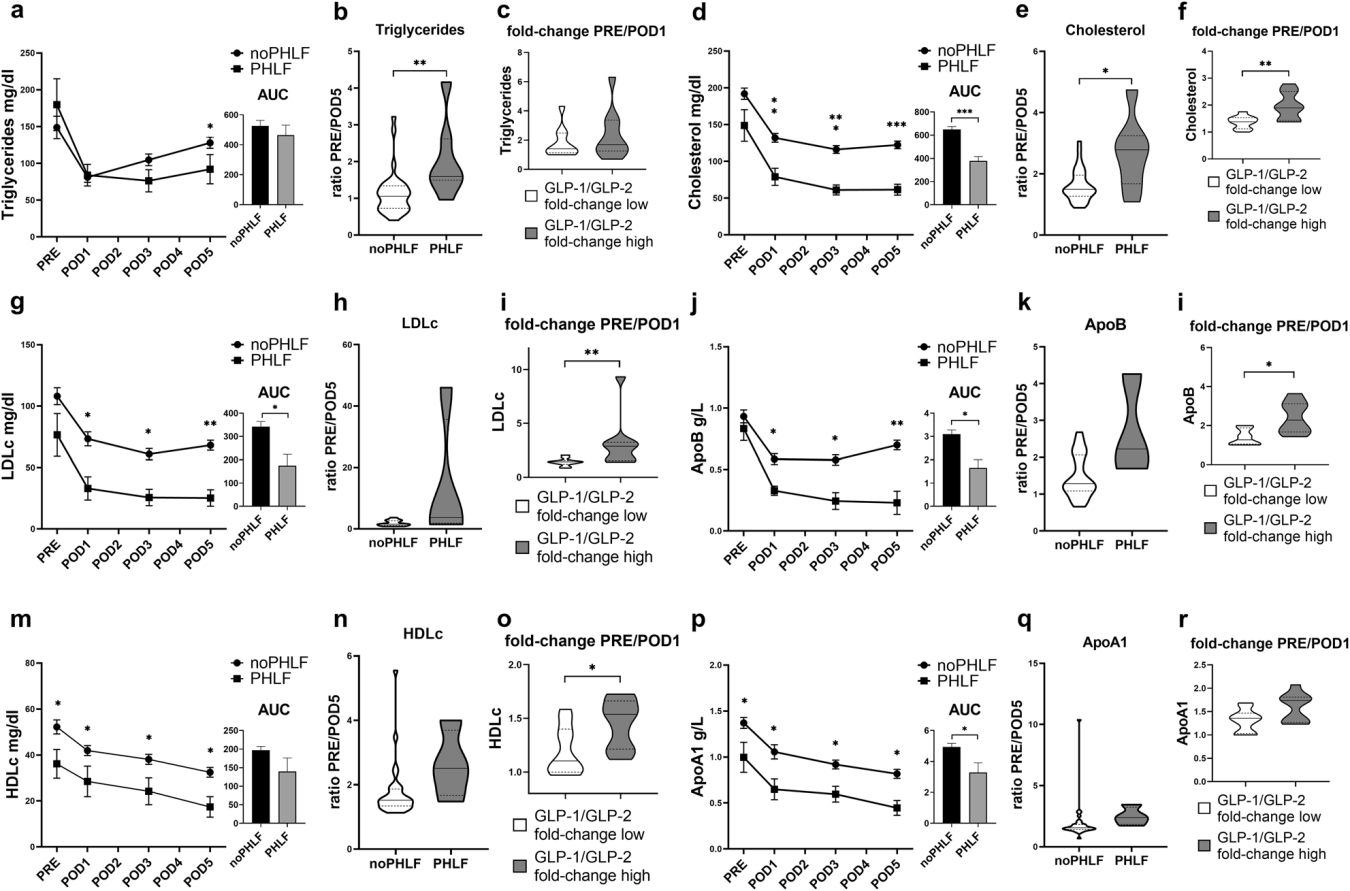
Perioperative plasma lipid parameter dynamics in PHLF. Graphs and inserted columns with error-bars depict perioperative plasma concentration dynamics and AUC comparison for triglycerides (**a**), total cholesterol (**d**), LDLc (**g**), ApoB (**j**), HDLc (**m**) and ApoA1 (**p**). Violine blots represent fold-changes of plasma lipid parameter after 5 postoperative days, (**b**, **e**, **h**, **k**, **n**, **q**); Delineated are associations of postoperative changes of plasma lipid parameters with the postoperative alterations of GLP1/GLP-2 ratios in representative groups of most extreme postoperative change of the GLP-1/GLP-2 ratio (GLP-1/GLP-twofold-change low/high, n = 10, respectively) (**c**, **f**, **i**, **l**, **o**, **r**); horizontal and dotted lines for means and quartiles; Mann–Whitney-*U* test; *LDLc* low-density lipoprotein cholesterol, *ApoB* apolipoprotein B, *HDLc* high- density lipoprotein cholesterol, *ApoA1* apolipoprotein A1; *p < 0.05; **p < 0.005; ***p < 0.0005.

Comparing baseline lipid metabolism parameters preoperatively between patients with and without PHLF, we found decreased levels of HDLc and ApoA1 in PHLF. Triglycerides, cholesterol, LDLc and ApoB level were seen unaltered. In both groups all lipid parameters declined postoperatively (Fig. 4a + d + g + j + m + p, Supplementary Materials & Methods Fig. S5).

When examining changes in plasma lipid levels until POD5, expressed as ratios to illustrate alterations relative to the baseline levels, only triglycerides and total cholesterol appeared to significantly decrease in PHLF. This decrease is presumably due to either increased consumption or exhausted mobilization/production in PHLF (Fig. 4b + e + h + k + n + q). Examining the time course, delineated as AUC, we did not observe any differences in triglyceride levels within the first 5 days postoperatively (Fig. 4a, Supplementary Materials & Methods Table S2).

Is has been reported previously, that GLP-1 and GLP-2 exhibit inverse effects on plasma lipid levels. To evaluate the balance of GLP-1 and GLP-2 on plasma lipid parameters, we integrated patients, who displayed the most extreme postoperative changes in the GLP-1/GLP-2 ratio, into a GLP-1/GLP-2 “high” and “low” group, and compared postoperative changes of lipid parameters, in order to delineate the combination of GLP effects on plasma lipid parameters postoperatively. We saw, that within patients who had a more pronounced postoperative decrease in the GLP-1/GLP-2 ratio, a significant association with a stronger decrease of lipid parameter (except triglycerides) could be observed (Fig. 4c + f + i + l + o + r). Given the fact that GLP-1 has a lipid lowering and GLP-2 a lipid increasing effect, these observations suggest a postoperative decline of GLP-1 and/or increase of GLP-2 levels may reflect a compensatory mechanism to maintain lipid homeostasis during liver regeneration. In patients with PHLF, postoperative GLP-2 AUCs trended to correlate with triglyceride AUCs (R = 0.750) and inversely with total cholesterol AUCs (R = − 0.821) (Supplementary Materials & Methods Table S5), suggesting that up to a certain point in PHLF, plasma lipid concentrations are linked to GLP-2 plasma levels.

## Discussion

With this study we were able to explore the time course of GLP-1 and GLP-2 in a yet unexplored clinical context, which expands our current knowledge of their “incretin” properties of regulating energy substrate resorption, trafficking and homeostasis postprandially^15^. We uncovered GLP-1 and GLP-2 being dynamically regulated in human liver regeneration after hepatic resections, independent of oral food intake. Intriguingly, when liver regeneration happens to be impaired and PHLF occurred, postoperative GLP-1 and GLP-2 plasma concentrations were subjected to even more pronounced changes, compared to the dynamics seen in patients who did not develop PHLF. We could present, that these variations in plasma GLP levels were not solely associated with the volume of lost liver tissue, or the development of severe postoperative morbidity, as could have been assumed, considering previous studies which reported GLP-1 elevation in patients with sepsis^16^. Our data delineated a pronounced increase in GLP-2 plasma levels, with a concomitant distinctive decline in GLP-1 concentrations in PHLF patients. This converse postoperative dynamics were unexpected, given the fact that GLP-1 and GLP-2 are released in parallel, in equimolar concentrations from enteroendocrine L-cells, triggered by the same stimulus^15^.

We initially hypothesized that postoperative alterations of GLP-1 and GLP-2 concentration might be caused by changes in their degradation kinetics. DPP4 is an exopeptidase, expressed on the cell surface of many different cell-types including hepatocytes^17^, but it also occurs in an enzymatically active soluble form, cleaved from the cell surface. Interestingly degradation of GLPs happens so fast after secretion, that only 25% of active GLP-1 leaves the gut and another 50% is degraded in the portal venous system with only 10–15% leaving the liver intact^15^, underlining the major role of the liver in GLP clearance.

Given the fact, that we detected hepatic DPP4 expression on hepatocytes, in immunohistochemical staining predominantly on the bile canaliculi directed surface in the perivenous portal zone, and not in the sinusoidal space, we speculated that the distinctive degradation of GLPs on their passage through the liver is facilitated via the soluble DPP4 in the portal circulation and not by the membrane bound form. Strikingly, we observed a significant decrease of DPP4 postoperatively in general, but the resection extend solely did not account for any differences in plasma DPP4 level, assuming a different primary source of soluble DPP4 e.g. adipose, visceral adipose tissue and also hematopoietic cells have been discussed in the literature^18^. Acknowledging these facts, postoperative GLP-1 and GLP-2 alterations in PHLF are not explainable solely by circulating DPP4 level.

Since GLP-1 and GLP-2 plasma level dynamics cannot be explained by perioperative changes of their degrading enzyme, we thought to focus on activators of GLP secretion, previously described. Bile acids are known to activate L-cells via binding to the extracellular G-protein-coupled bile acid receptor TGR5, leading to GLP secretion^19^. During liver regeneration bile acids play a critical role by inducing the cell-cycle key regulator FoxM1b^20^ as well as in the regulation of multiple metabolic pathways via interaction with the farnesoid X receptor (FXR)^21^. Vice versa, GLP-2 regulates hepatocyte intracellular bile acid synthesis and export into the bile fluid and systemic circulation^22^. In contrast to our observations, it has been reported, that as a consequence of liver resection, plasma bile acid level increase proportionally to the extent of resected liver volume^23^. In PHLF patients, plasma bile acid concentrations were not subjected to alterations, and did not correlate with GLP levels postoperatively, attributing them to a minor role in promoting GLP secretion in PHLF. It has to be mentioned, that the complexity of bile acid metabolism and their variety of involved regulative pathways including cells of the entero-hepatic axis with luminal and basolateral exposure to a plethora of bile acid derivates, might not be accurately reflected by the simple correlation analysis of GLPs and total bile acid plasma concentrations.

Moreover, L-cell activation via IL-6 might be of greater relevance^24^, particularly in situations of significant trauma as hepatic resection. IL-6 is a proinflammatory cytokine with a crucial function in the priming phase of liver regeneration^25^. It is secreted by several cell types like fibroblasts, immune cells and endothelial cells upon various signals, due to activation via tumour necrosis factor (TNF)-α or Interleukin-1 in the acute phase response^26^. Following liver resection, bacterial constituents like lipopolysaccharides (LPS) translocate into the portal venous system, resulting in toll-like receptor (TLR)-4 mediated activation of liver resident Kupffer-cells to induce TNF-α release, leading to a rapid and pronounced IL-6 secretion within the liver^27^.

In that context, as expected, we observed a postoperative rapid and significant increase in circulating IL-6, but differences in IL-6 levels regarding the resection extent could not be documented. If the volume of resected liver tissue is connected to IL-6 alterations, or associated with organ dysfunction is controversially discussed throughout the literature^28^. IL-6 is a potent activator of L-cells^24^. In our cohort PHLF was associated with elevated IL-6 level in the perioperative course, likely explaining our observed correlations with plasma GLP-2 levels during the perioperative observational period. Interestingly, GLP secretion is also stimulated via basolateral contact of LPS with the TLR-4^29^ per se and both, GLP-1 and GLP-2 are known to reduce gut permeability to prevent further bacterial translocation^29,30^, strongly suggesting a attenuating role in hepatic inflammation.

Given our current knowledge, liver regeneration is crucially dependent on lipid supply^2–4^. Both, GLP-1 and GLP-2 facilitate critical, but opposing regulatory effects in lipid absorption, trafficking and metabolism. In fact, GLP-1 mediates a decrease of chylomicron production and reduces plasma triglyceride levels, termed “fasting dyslipidaemia” in different clinical contexts^31,32^. On the contrary, GLP-2 is known to increase postprandial triglyceride-rich chylomicrons primarily via upregulation of intestinal apoB48 synthesis and release from preformed stores, confirmed in mice and hamsters^31,33^ as well as in humans^34^. Also by enhancing intestinal lymphatic flow, net chylomicron and triglyceride output is increased^35^. Additionally, GLP-2 acts on hepatic VLDL production and lipogenesis, as well causing elevated plasma triglyceride level and consecutively leading to hepatic steatosis^7^. Lipogenic effects are not restricted to the gut-liver axis, Ejarque et al. reported an elevation of hormone sensitive lipase and adipocyte triglyceride lipase expression in subcutaneous and visceral adipose tissue after GLP-2 administration in mice, indicating a lipid mobilising effect^36^.

The opposing postoperative GLP-1 and GLP-2 dynamics in PHLF, as we have documented, presumably mirror a pro lipogenic GLP constellation in terms of massive energy requirements of the regenerating liver. In fact, this might explain why GLP-1 administration in partially hepatectomised rats affect liver regeneration negatively^11^, while regeneration in mice, receiving GLP-2 prior to partial hepatectomy was improved^10^.

Given the fact that we observed the strongest pro-lipogenic GLP-1/GLP-2 constellation in patients with more pronounced postoperative declines in parameters of lipid metabolism, we hypothesise that GLPs are regulated according to the increased energy demands, representing a rescue signal in patients with impaired or exhausted liver regeneration. Interestingly, triglycerides did not display that association, suggesting that their homeostasis might still be maintained in an early stage after surgery. Of note, in patients with pre-existing hepatic steatosis, histological markers of liver inflammation, MASLD or obesity, GLP-1 baseline level were elevated, possibly reflecting a metabolic prerequisite, unfavourable for liver regeneration in that context.

At this point it has to be mentioned, that most of our knowledge about the biological actions of GLPs on lipid metabolism are derived from studies with long term GLP administration or animal gene knockout models, especially in the postprandial setting, as the scope of incretin research, up to date, has been focusing mainly on disease from metabolic syndrome. Given the fact, that systemic GLP actions seem to change during chronic exposure^31^, immediate or short notice effects might not be reflected in those studies and extrapolating their findings into our setting should be interpreted with caution. We must acknowledge several additional limitations to the findings of this study. Our study cohort comprises a relatively small number of patients. In light of ongoing discussions surrounding the redefinition of the threshold for statistical significance in biomedical research^37^, we opted for a significance level of p < 0.005 for preoperative plasma parameters in order to mitigate the risk of a type I error arising from limited statistical power. However, when interpreting our further exploratory data in the context of observed effects, we adhered to the conventional significance threshold of p < 0.05 to minimize the risk of a type II error.

Furthermore, the results of this study should be approached with caution due to the unequal distribution of liver diseases necessitating liver resection among groups, particularly in relation to the development of PHLF. Notably, preoperative elevations in γ-GT and AP levels in PHLF patients may be associated with cholestasis, which is more frequently observed in cases of intrahepatic and perihilar cholangiocarcinoma (iCCC/pCCC). Additionally, it is worth noting that no instances of post-hepatectomy liver failure (PHLF) occurred in patients who underwent minor resections, a factor that may have influenced both the results and their interpretations.

Another limitation pertains to our measurement of both isoforms of GLP-1 (7–36 and 9–36) and GLP-2 (1–33 and 3–33). It is worth noting that GLP-1 (9–36) and GLP-2 (3–33) are traditionally considered inactive isoforms. However, accumulating evidence suggests that they might exert distinct and partially agonistic biological effects^38,39^. We presented the dynamics of the total circulating GLP concentrations to infer secretory changes. It is important to mention that there is currently no available data regarding the plasma half-life times of these truncated isoforms, and alterations in their proportion within the total GLP range could occur during the postoperative period. Nevertheless, given that plasma levels of both total GLP-1 and total GLP-2 are subject to fluctuations postoperatively, our data offer evidence of secretory regulation during postoperative liver regeneration.

**Figure 5:**
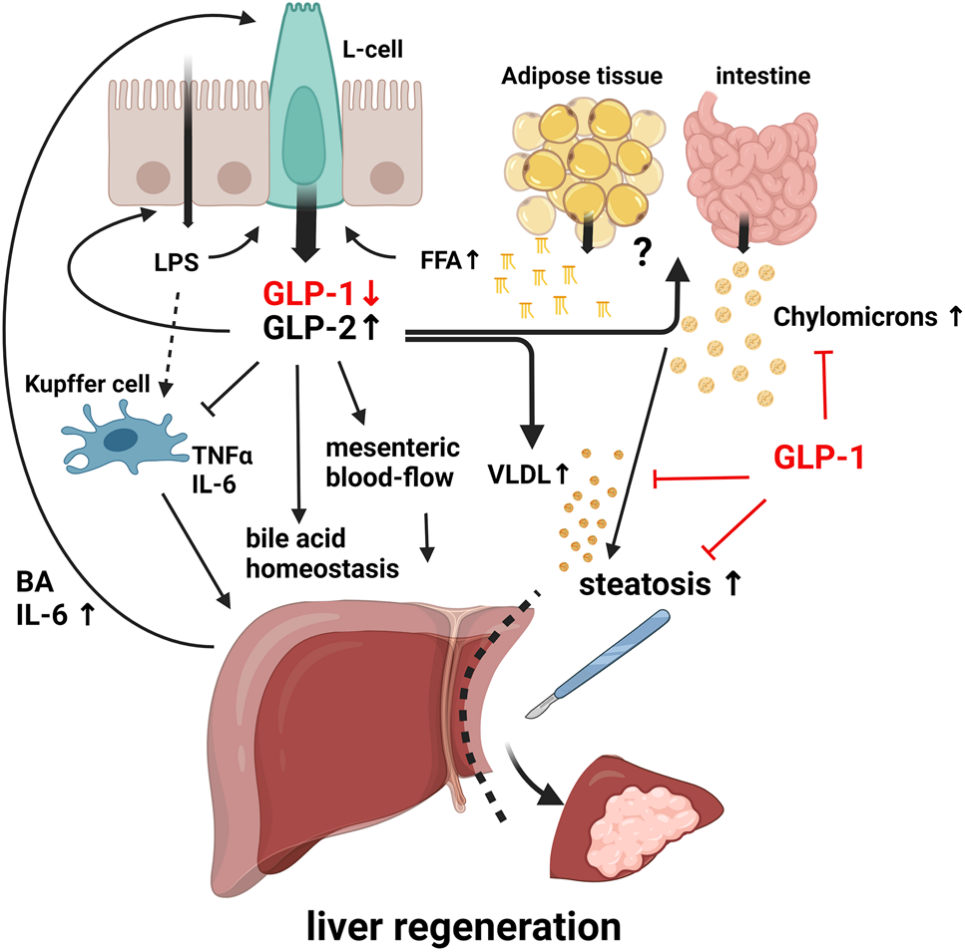
Potential effects of GLP-1 and GLP-2 during post-hepatectomy liver regeneration. During liver regeneration, GLP-1 and GLP-2 are secreted by enteroendocrine L-cells independently of nutritional stimuli, via IL-6 and BA-mediated basolateral contact with their corresponding receptors. BA levels are elevated in the remnant liver and plasma upon liver resection and comprise a mitogen for liver regeneration. GLP-2 facilitates effects on hepatic intracellular BA homeostasis. Reduction of the total portal venous cross-section after extensive liver tissue loss increases portal venous pressure, compromises gut barrier function, and increases bacterial translocation. Kupffer cells are activated by LPS via a TLR4-mediated pathway, leading to TNFα release and perpetuating IL-6 secretion from further activated Kupffer cells, endothelial cells, and hepatocytes. L-cells further release GLPs followed by activation of basolateral TLR4-LPS interaction. At the level of the gut barrier, GLP-2 decreases its permeability and additionally facilitates a blunting of macrophage activation, hence regulating hepatic inflammation. GLP-2 possibly contributes to liver regeneration by increasing mesenteric blood and lymphatic flow, conceivably by accelerating hepatic energy supply. During liver regeneration, the development of transient hepatic steatosis is required to meet increased energy demands. GLP-2 facilitates the release of triglyceride-rich ApoB48-containing chylomicrons and mobilizes lipid stores from the liver via increased VLDL secretion, presumably for lipid redistribution, whereas GLP-1 opposes these effects. Mobilization of peripheral lipids from adipose tissue during liver regeneration possibly involves a GLP-2 receptor-mediated mechanism, expressed in visceral and subcutaneous adipose tissue. Elevated postoperative GLP-2 concentrations in PHLF might comprise a rescue signal within the gut-liver axis, either to regulate hepatic inflammation or to attempt to restore diminishing energy supply to the liver during hepatic regeneration. *GLP-1* Glucagon-like peptide-1, *GLP-2* Glucagon-like peptide-2, *BA* bile acids, *IL-6* Interleukin-6, *TNFα* tumor necrosis factor α, *TLR4* toll-like receptor 4, *LPS* lipopolysaccharides, *VLDL* very low-density lipoprotein, *FFA* free fatty acids.

So far, it is unclear if endogenous GLP-1 and GLP-2 impact liver regeneration, but this study provides evidence that alterations of the endogenous GLP system seem to correlate with impaired liver regeneration. A recovering role in lipid metabolism seems likely, also Psichas et al. reported basolateral sensing of free fatty acids in L-cells^40^, which provides a hint towards a direct regulatory mechanism in facilitating lipid homeostasis. Additional GLP-2 mediated effects on liver regeneration are also considerable. An increase in mesenterial blood-flow via a nitric oxide (NO) dependant mechanism was described^41^, as well as a negative regulatory feedback loop involving gut permeability, bacterial translocation, activation of Kupffer-cell facilitated IL-6 release and consecutive GLP secretion (discussed above), or GLP-2 mediated blunting of inflammatory macrophage (M1) activation^42^ might also contribute and provide intriguing perspectives on the gut-liver axis in the matter of post hepatectomy liver regeneration (Fig. 5).

If these mechanisms could be addressable as therapeutic targets, has to be clarified in further studies. Our study provides a new body of evidence for endogenous GLPs being involved in human liver regeneration and offer a promising new perspective to pursue potentially addressable targets to prevent, or even treat PHLF.

## Material and methods

In this prospective observational study, 46 patients who underwent primary liver resection or partial hepatectomy after future remnant liver augmentation (double vein embolization (DVE) or associated liver partition with portal vein ligation for staged hepatectomy (ALPPS)), for malignant and benign hepatic tumours, between August 2019 and October 2021, in a single centre, were included. Resection extent was graded after the Brisbane 2000 nomenclature as major (≥ 3 anatomical liver segments) or minor (< 3 anatomical liver segments) resection^43^. Patients suffering from type II diabetes mellitus stopped taking oral antidiabetics of any kind (including DPP4 inhibitors), at least 24 h prior to the operation. This study was approved by the institutional Ethics Committee (Niederoesterreichische Ethikkommission GS-1-EK-4/568-2018) and conducted in accordance with the principles of the Declaration of Helsinki and the guidelines for Good Scientific Practice. All patients gave written informed consent.

### Definition of outcome parameters

Clinical endpoints where set for post-hepatectomy liver failure (PHLF) and postoperative morbidity. The International Study Group of Liver Surgery (ISGLS) classification of PHLF was applied. Briefly, elevated serum bilirubin level (> 1.2 mg/dl) and a prolonged prothrombin time (< 70%) on, or beyond POD5, according to the threshold level of the local laboratory defines PHLF^44^. In case of mortality within 5 postoperative days, patients were defined as PHLF when bilirubin and prothrombin time were altered according to the forementioned classification.

The classification of Dindo et al. for postoperative morbidity was used (grade I–V). Given multiple complications, the most severe one was taken into account. Patients who required invasive management (surgery, radiologic or endoscopic intervention) or died from postoperative complication where issued as severe morbidity (grade III–V)^45^.

### Plasma preparation and measurement

Blood was collected after overnight fasting prior to surgery (PRE), on POD1 and POD5. Blood was drawn into prechilled tubes containing EDTA, a DPP4-inhibitor (10 µmol/l, Sigma Aldrich, St. Louis, MO) and Aprotinin (500 KIU/ml, Sigma Aldrich, St. Louis, MO) for protease inhibition, according to established protocols^46^. Plasma preparation was carried out as described previously^47^. In brief, withdrawn blood was placed on ice immediately and centrifuged within 15 min at 1000×*g* at 4 °C for 10 min. Subsequently, the supernatant was collected and centrifuged repeated at 10.000×*g* at 4 °C for 10 min to improve purification from cell detritus and platelets. Plasma was stored in aliquots at − 80 °C for further use. Aliquots thawed more than once have not been used for analysis.

Commercially available enzyme-linked immunosorbent assays (ELISA) were used for determination of total GLP-1 (7–36 and 9–36) (ALPCO, Salem, NH), GLP-2 (1–33 and 3–33) (Merck Millipore, Burlington, MA) and DPP4 (R&D Systems, Minneapolis, MN) plasma concentrations and were carried out according to the manufacturer’s instructions.

Routine laboratory parameters such as alanine aminotransferase (ALT), aspartate aminotransferase (AST), γ-glutamytransferase (γ-GT), alkaline phosphatase (AP), bilirubin, prothrombin time, bile acids, interleukuin-6 (IL-6), triglycerides, total cholesterol, low-density lipoprotein cholesterol (LDLc), high-density lipoprotein cholesterol (HDLc), apolipoprotein A1 (ApoA1) and apolipoprotein B (ApoB) were measured perioperatively in fasting plasma.

### Histological analysis

To determine the stage of an underlying liver disease, tumour distant liver tissue from the resection specimens were analysed by 2 trained clinical pathologists, blinded for patient’s clinical outcome. Two µm slices of formalin-fixed paraffin-embedded (FFPE) tissue were dyed with hematoxillin/eosin (H&E) for determination of metabolic dysfunction-associated fatty liver disease (MASLD) and chromotrope-anilin blue (CAB) trichrome staining for fibrosis assessment. MASLD was evaluated applying the (NAFLD) activity score (NAS), by allocating points for histological findings of steatosis, hepatocyte ballooning and inflammation activity^48^. Summarized points ≥ 3 were defined as MASLD according to the Delphi consensus statement on fatty liver disease nomenclature^49^. Fibrosis was evaluated applying the Kleiner classification (f0–f4)^50^. Absent fibrosis (f0) was compared to histological findings of fibrosis of any stage (f1–f4). Inflammation activity (sum of hepatocyte ballooning and acinary activity) was dichotomized: mild (≤ 1 points) or severe (≥ 2 points) activity^51^.

### Statistical analysis

Analysis was based on non-parametric tests due to low sample sizes in rare clinical outcome groups and to receive more robust information regarding outliers. For comparison of means the Mann–Whitney-*U* Test was applied. For calculations of differences in dynamics over time the Wilcoxon signed-rank test for paired samples was used. Correlation analysis applying the Spearman test were carried out. *p* < 0.05 was considered statistically significant. For determination of concentration over time, Area Under the Curve (AUC) analysis was performed. In the explorative analysis p < 0.05 was considered statistically significant, whereas for preoperative laboratory parameters the level of significance was set to p < 0.005^37^. Statistical analysis were computed using SPSS® version 20 (IBM, Armonk, NY) and Graphpad Prism 8.0.2 (GraphPad Software Inc., San Diego, CA).

### Supplementary Information

Supplementary Information.

## Data Availability

The datasets generated and analysed during the current study are not publicly available due to ethical consideration, but are available from the corresponding author on reasonable request.

## Abbreviations

LR: Liver resection
GLP-1: Glucagon-like peptide-1
GLP-2: Glucagon-like peptide-2
PHLF: Post-hepatectomy liver failure
IL-6: Interleukin-6
MASLD: Metabolic dysfunction-associated liver disease
HDLc: High-density lipoprotein cholesterol
LDLc: Low-density lipoprotein cholesterol
VLDL: Very low-density lipoprotein
ApoA1: Apolipoprotein A1
ApoB: Apolipoprotein B
DPP4: Dipeptidyl peptidase 4
AUC: Area under the curve
