# Supplementary material for: Glucagon-like peptide-1 and glucagon-like peptide-2 regulation during human liver regeneration
Source: Sci Rep. 2023 Sep 25;13:15980. doi: 10.1038/s41598-023-43283-8 (PMC10519971; doi:10.1038/s41598-023-43283-8)
Supplement: Supplementary file 1 — Supplementary Information. [file 41598_2023_43283_MOESM1_ESM.pdf]

Supplementary Materials & Methods

Glucagon-like peptide-1 and glucagon-like peptide-2 regulation during human liver regeneration

Authors

Markus Ammann [1,2]; tellammann@gmail.com

Jonas Santol [3]; tellsantol@gmail.com

David Pereyra [2]; tellpereyra@gmail.com

Tamara Kalchbrenner [4]; tamara.kalchbrenner@wienerneustadt.lknoe.at

Tanja Wuerger [4]; twuerger@live.de

Johannes Laengle [2]; johannes.laengle@meduniwien.ac.at

Rory L. Smoot [5]; smoot.rory@mayo.edu

Wolfgang Hulla [4]; wolfgang.hulla@wienerneustadt.lknoe.at

Friedrich Laengle [1]; friedrich.laengle@wienerneustadt.lknoe.at

Patrick Starlinger [2,5]; tellstarlinger@gmail.com

Table of contents

Fig. S1. Correlations of GLP-1 and GLP-2 plasma level at perioperative timepoints in patients with or without PHLF 2

Fig. S2. GLP-1 and GLP-2 plasma concentrations in co-morbidities and histopathological findings of tumour distant liver tissue 3

Supplementary method 4

Fig. S3. Representative image of immunohistochemistry staining for DPP4 4

Fig. S4. Correlations of bile acids, IL-6 and DPP4 with GLP-1 and GLP-2 5

Fig. S5. Plasma lipid parameter level at perioperative timepoints 6

Supplementary tab. S1. 7

Supplementary tab. S2. 8

Supplementary tab. S3 9

Supplementary tab. S4 10

Supplementary tab. S5. 11

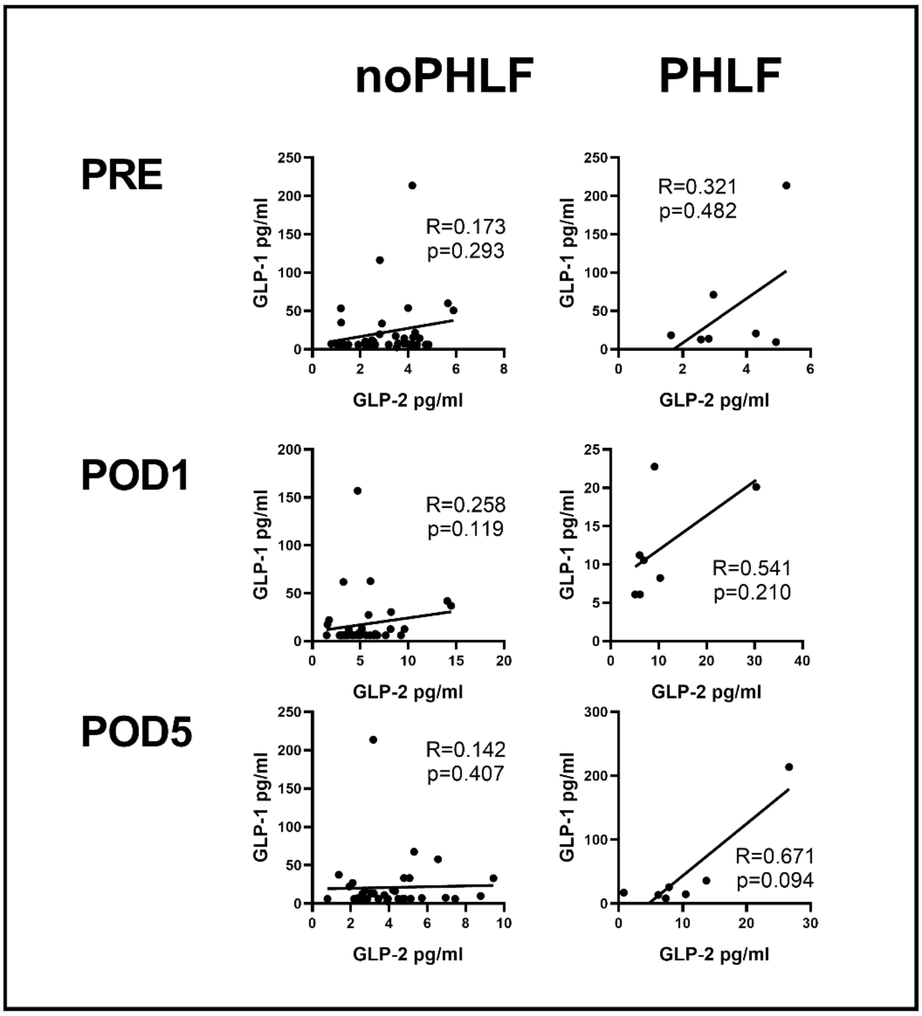

Fig. S1. Correlations of GLP-1 and GLP-2 plasma level at perioperative timepoints in patients with or without PHLF. Presented are scatterplots with regression lines. Two-sided Spearman correlation was performed for specific timepoints in patient groups.

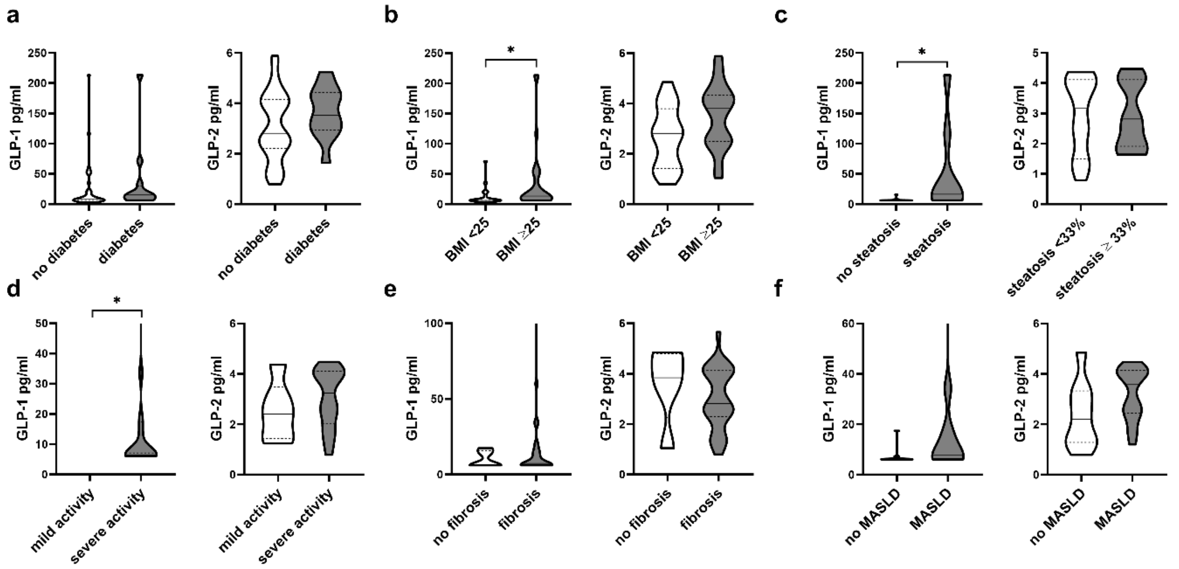

Fig. S2. GLP-1 and GLP-2 plasma concentrations in co-morbidities and histopathological findings of tumour distant liver tissue. Presented are comparisons of GLP-1 and GLP-2 level regarding the presence of diabetes mellitus (a), between patients with a normal and elevated BMI (b), regarding steatosis of a greater extent than 33% of hepatocytes (c), inflammatory activity (mild activity: ≤2pts, severe activity: >2pts regarding hepatocyte ballooning and inflammation) (d), the presence of fibrosis of any grade f1-4 (e), and MASLD (f). Violine-blots with mean and quartiles; Man-Whitney-U test; BMI, body mass index, MASLD, metabolic-dysfunction associated liver disease; *p<0.05.

Supplementary method

Immunohistochemistry staining of DPP4: formalin- fixated paraffin- embedded resection specimens were sliced into 2-3µm sections. Deparaffinization, heating and incubation with the monoclonal DPP4 antibody, TrueMAB™ (1:500, OTI11D7, Thermofisher Scientific, Waltham, MA) were performed automatically using VENTANA BenchMark ULTRA (Roche Diagnostics, Mannheim, Germany). Antigen- antibody reaction was visualized using Ventana UltraView Universal DAB Detection Kit (Roche Diagnostics, Mannheim, Germany). Counterstaining was conducted with hemalum-acetic acid- ddH_2O followed by bluing. Image was interpreted by a trained clinical pathologist.

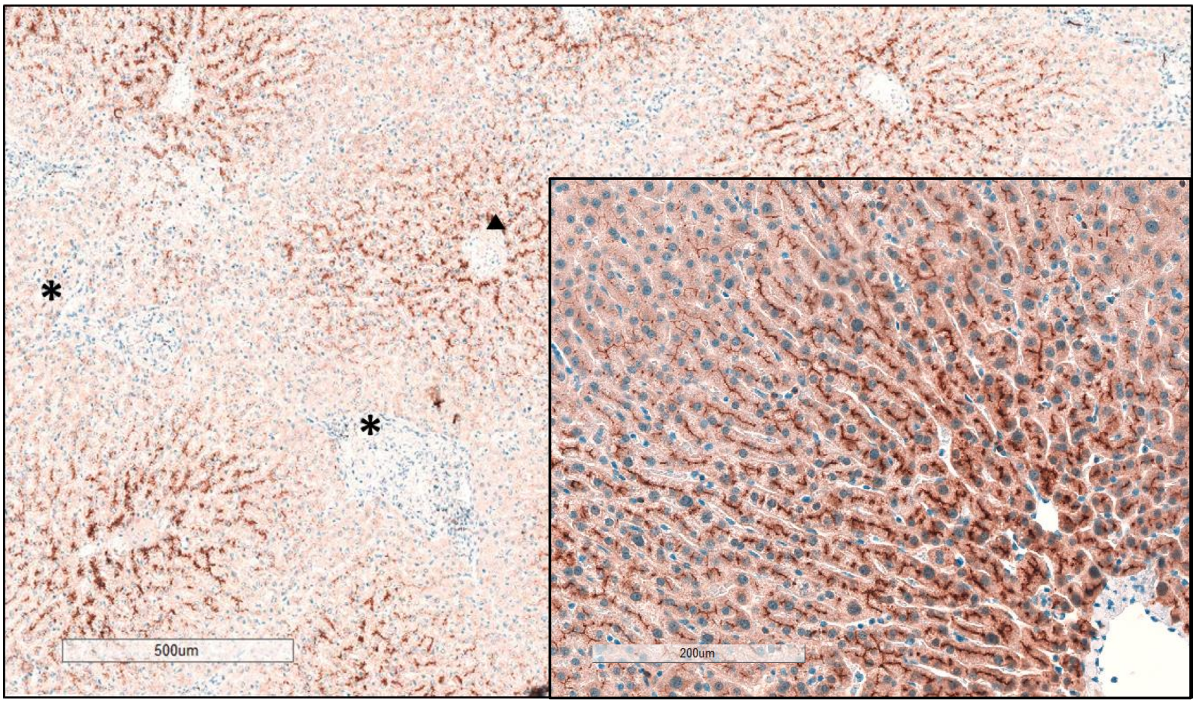

Fig. S3. Representative image of immunohistochemistry staining for DPP4. DPP4 expression in the portal fields of a liver lobule; Magnification of one peri-central field (portal zone 3) displaying DPP4 expression in the bile canaliculi between lines of hepatocytes and missing DPP4 staining in the sinusoids or blood vessels; central vein (black triangle); portal triad (black asterisks). Scale bars for 500µm (main image) and 200µm (inserts) are given.

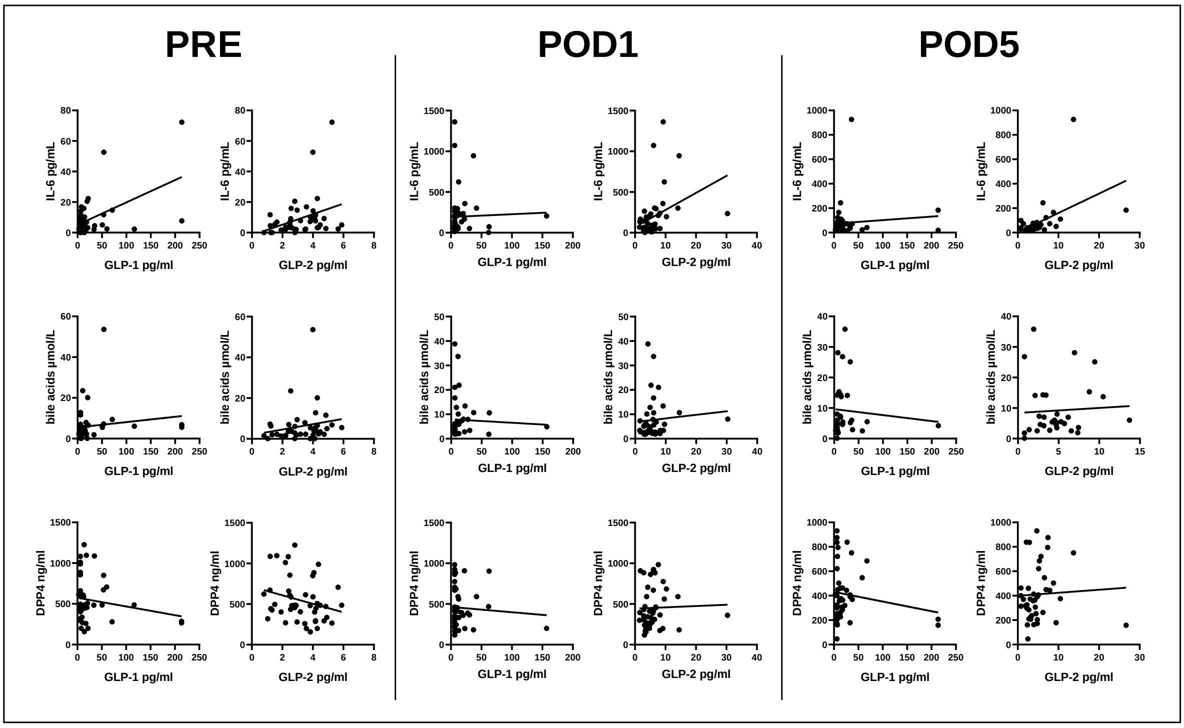

Fig. S4. Correlations of bile acids, IL-6 and DPP4 with GLP-1 and GLP-2. Scatterplots with regression line for plasma concentration correlations of indicated parameters at perioperative timepoints.

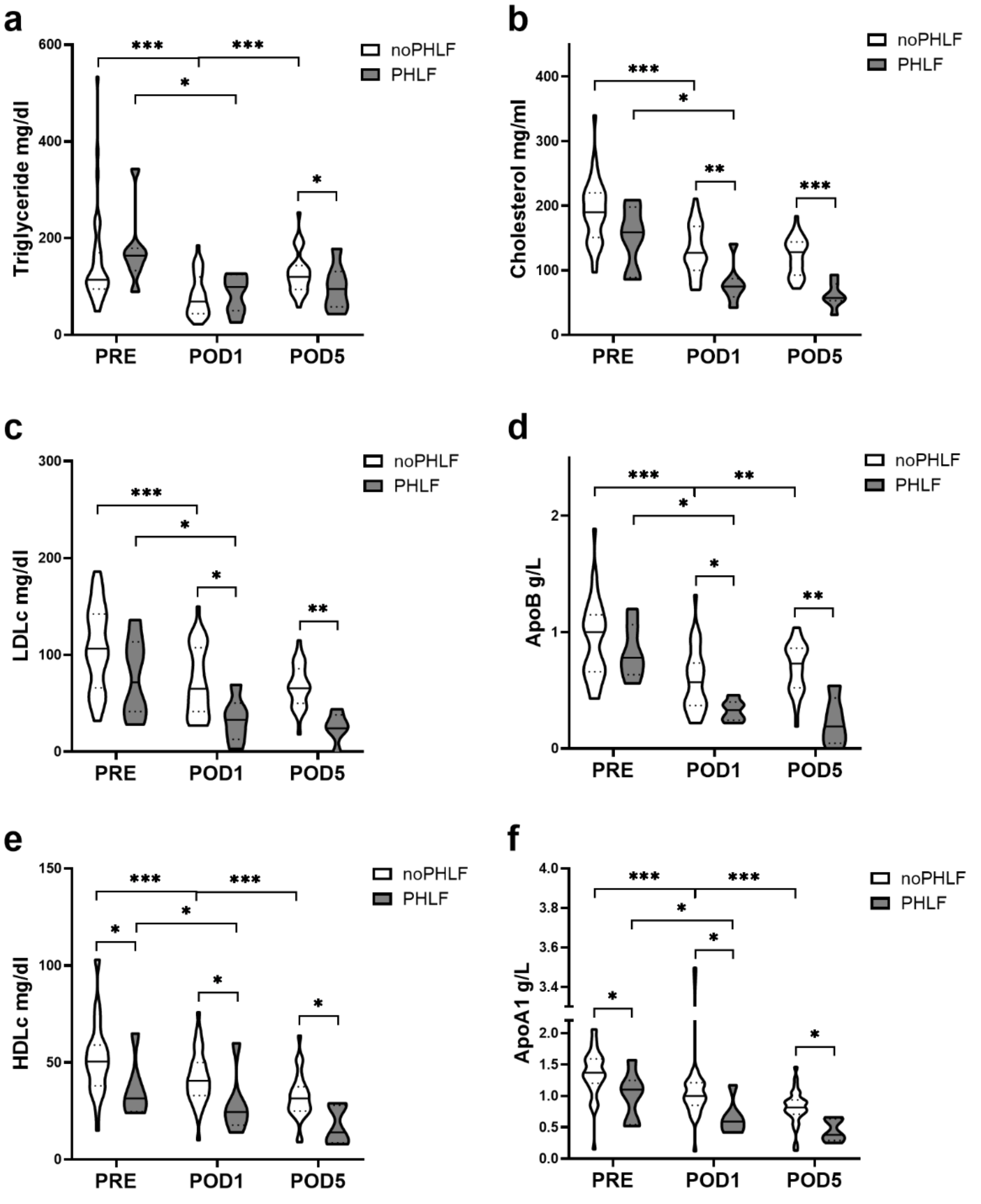

Fig. S5. Plasma lipid parameter level at perioperative timepoints. Violine-blots display plasma lipid parameter level within groups, for triglycerides (a), total cholesterol (b), LDLc (c), HDL (d), ApoA1 (e)and ApoB (f) (Mann-Whitney-U test) and changes between timepoints (Wilcoxon signed-rank test). horizontal and dotted lines for means and quartiles; LDLc, low-density lipoprotein cholesterol, ApoB, apolipoprotein B, HDLc, high-density lipoprotein cholesterol; ApoA1, apolipoprotein A1; *$p<0.05$; **$p<0.005$; ***$p<0.0005$.

Supplementary tab. S1. Area under the curve (AUC) analysis of resection extend (minor vs. major liver resection). Patients with PHLF and morbidity ≥3 excluded.

	minor		major		
	**n**	**Median (range)**	**n**	**Median (range)**	**p-value**
**GLP-1 (pg/ml)**	22	35.46 (22.10- 926.70)	8	57.02 (30.51- 318.80)	0.126
**GLP-2 (pg/ml)**	21	20.27 (9.17- 51.14)	10	29.21 (8.22- 36.29)	0.820
**Bile acids (µmol/l)**	18	21.95 (7.00- 81.20)	7	49.45 (24.70- 91.30)	0.845
**IL-6 (pg/ml)**	18	435.10 (79.00- 3539.00)	10	484.90 (152.60- 1676.00)	0.774
**DPP4 (ng/ml)**	21	1871.00 (816.90 - 4561.00)	10	1900.00 (818.10- 4051.00)	**0.028**
**Triglycerides (mg/dl)**	19	517.50 (317.00- 1236.00)	10	378.00 (262.0- 792.5)	**0.014**
**Total cholesterol (mg/dl)**	23	651.00 (436.00-1033.00)	10	538.75 (372.5- 757.0)	0.286
**LDLc (mg/dl)**	17	333.50 (169.00- 583.00)	10	316.50 (124.5- 493.0)	0.970
**ApoB (g/l)**	20	3.09 (1.08- 5.40)	10	2.74 (1.665- 4.435)	0.494
**HDLc (mg/dl)**	21	194.00 (127.50- 348.50)	10	170.50 (49.5- 325.0)	0.441
**ApoA1 (g/l)**	21	5.19 (3.88- 7.11)	10	3.70 (0.655- 8.365)	0.093

AUC analysis from preoperative (PRE) to POD5. Given are AUCs medians; values in parenthesis indicate rages; DPP4, dipeptidyl peptidase-4; GLP-1, glucagon like peptide-1; GLP-2, glucagon-like peptide-2; IL-6, Interleukin-6; LDLc, low-density lipoprotein cholesterol; ApoB, apolipoprotein B; HDLc, high-density lipoprotein cholesterol; ApoA1, apolipoprotein A1; Man-Whitney-U test.

Supplementary tab. S2. GLP-1, GLP-2 and DPP4 plasma concentrations.

		noPHLF		PHLF		
		**n**	**Median (range)**	**n**	**Median (range)**	**p**
GLP-1 (pg/ml)						
	PRE	39	7.26 (2.64- 213.71)	7	18.47 (9.56- 213-71)	**0.030**
	POD1	38	6.10 (5.94- 156.98)	7	10.55 (6.10- 22.76)	0.451
	POD5	36	7.42 (2.97- 213.71)	7	17.15 (8.25- 213.71)	**0.041**
GLP-2 (pg/ml)						
	PRE	39	3.18 (0.79- 5.89)	7	2.96 (1.64- 5.25)	0.383
	POD1	38	4.75 (1.50- 14.47)	7	6.89 (5.10- 30.36)	**0.008**
	POD5	36	3.83 (0.79- 9.44)	7	7.87 (0.79- 26.67)	**0.006**
DPP4 (ng/ml)						
	PRE	39	481.20 (157.70- 1087.00)	7	506.90 (265.80- 1226.00)	0.788
	POD1	38	359.90 (118.60- 983.00)	7	454.20 (198.70- 924.40)	0.234
	POD5	36	361.45 (45.93- 930.10)	7	443.60 (157.70- 794.40)	0.468

Median plasma concentrations at indicated timepoints; values in parenthesis display ranges; GLP-1, glucagon like peptide-1; GLP-2, glucagon-like peptide-2; DPP4, dipeptidyl peptidase-4; Man-Whitney-U test.

Supplementary tab. S3. Area under the curve (AUC) analysis**.**

	noPHLF		PHLF		
	**n**	**Median (range)**	**n**	**Median (range)**	**p**
**GLP-1 (pg/ml)**	35	38.09 (22.10- 926.70)	7	65.47 (38.67- 591.20)	0.114
**GLP-2 (pg/ml)**	35	20.23 (7.3- 57.21)	7	43.05 (28.87- 79.94)	**<0.001**
**Bile acids (µmol/l)**	30	26.13 (7.00- 156.70)	5	77.10 (13.95- 203.20)	0.299
**IL-6 (pg/ml)**	32	375.15 (79.0- 3539.0)	5	967.90 (646.50- 4535.00)	**0.005**
**DPP4 (ng/ml)**	35	1727.50 (816.9- 4750.0)	7	2179.00 (945.1- 4359.0)	0.323
**Triglycerides (mg/dl)**	34	495.00 (258.5- 1236.0)	7	384.00 (324- 726)	0.444
**Total cholesterol (mg/dl)**	38	648.75 (360.5- 1033.0)	7	374.50 (269.0- 553.5)	**<0.001**
**LDLc (mg/dl)**	32	333.00 (124.5- 627.5)	4	197.25 (50.0- 256.0)	**0.012**
**ApoB (g/l)**	34	2.99 (1.08- 5.40)	4	1.70 (0.75- 2.46)	**0.005**
**HDLc (mg/dl)**	36	191.75 (49.5- 348.5)	4	115.25 (86.0- 241.5)	0.135
**ApoA1 (g/l)**	36	4.95 (0.66- 8.37)	4	3.14 (1.93- 4.93)	**0.025**

AUC analysis from preoperative (PRE) to POD5. Given are AUCs medians; values in parenthesis indicate rages; DPP4, dipeptidyl peptidase 4; GLP-1, glucagon like peptide-1; GLP-2, glucagon-like peptide-2; IL-6, Interleukin-6; LDLc, low-density lipoprotein cholesterol; ApoB, apolipoprotein B; HDLc, high-density lipoprotein cholesterol; ApoA1, apolipoprotein A1; Man-Whitney-U test.

Supplementary tab. S4. Area under the curve (AUC) analysis of postoperative morbidity according to the Dindo classification. PHLF patients excluded.

	Morbidity <3		Morbidity ≥3		
	**n**	**Median (range)**	**n**	**Median (range)**	**p**
**GLP-1 (pg/ml)**	31	41.89 (22.10- 926.70)	5	63.66 (30.51- 591.20)	0.288
**GLP-2 (pg/ml)**	31	19.56 (8.22- 51.14)	5	37.35 (7.20- 57.21)	0.262
**Bile acids (μmol/l)**	25	25.45 (7.0- 91.3)	5	68.10 (15.25- 156.7)	0.355
**IL-6 (pg/ml)**	28	354.6 (79.0- 3539.0)	4	669.40 (323.7- 2477.0)	0.361
**DPP4 (ng/ml)**	31	1674.00 (816.9- 4561.0)	5	2119.50 (913.0- 4750.0)	0.690
**Triglycerides (mg/dl)**	29	511.00 (262.0- 1236.0)	5	383.00 (258.5- 611.5)	0.179
**Total cholesterol (mg/dl)**	33	651.00 (372.5- 1033.0)	5	422.50 (360.5- 749.0)	0.399
**LDLc (mg/dl)**	27	333.50 (124.5- 583.0)	5	247.00 (183.0- 627.5)	1.000
**ApoB (g/l)**	30	3.05 (1.08- 5.40)	4	2.00 (1.90- 3.56)	0.131
**HDLc (mg/dl)**	31	192.00 (49.5- 348.5)	5	144.00 (132.5- 236.0)	0.348
**ApoA1 (g/l)**	31	5.00 (0.66- 8.37)	5	3.56 (3.16- 5.60)	0.175

AUC analysis from preoperative (PRE) to POD5. Given are AUCs medians; values in parenthesis indicate rages; DPP4, dipeptidyl peptidase-4; GLP-1, glucagon like peptide-1; GLP-2, glucagon-like peptide-2; IL-6, Interleukin-6; LDLc, low-density lipoprotein cholesterol; ApoB, apolipoprotein B; HDLc, high-density lipoprotein cholesterol; ApoA1, apolipoprotein A1; Man-Whitney-U test.

Supplementary tab. S5. Spearman correlation of areas under the curv (AUC) for GLP-1, GLP-2, triglycerides and total cholesterol

		AUC GLP-1		AUC GLP-2	
		p-value	R	p-value	R
Triglycerides AUC	noPHLF	0.136	0.275	0.182	0.238
	PHLF	0.119	0.643	0.052	0.750
total Cholesterol AUC	noPHLF	0.442	-0.136	0.749	-0.056
	PHLF	0.071	-0.714	**0.023**	**-0.821**

GLP-1, glucagon-like peptide-1; GLP-2, glucagon like peptide-2.
